# Overexpression of KIF20A confers malignant phenotype of lung adenocarcinoma by promoting cell proliferation and inhibiting apoptosis

**DOI:** 10.1002/cam4.1710

**Published:** 2018-08-13

**Authors:** Xia Zhao, Lei‐lei Zhou, Xiaoyou Li, Jie Ni, Ping Chen, Rong Ma, Jianzhong Wu, Jifeng Feng

**Affiliations:** ^1^ The Affiliated Cancer Hospital of Nanjing Medical University Jiangsu Cancer Hospital Jiangsu Insititute of Cancer Research Nanjing Jiangsu China; ^2^ Department of Oncology First People's Hospital of Yancheng Fourth Affliated Hospital of Nantong University Yancheng Jiangsu China; ^3^ Department of Oncology The Affliated Huai'an No. 1 People's Hospital of Nanjing Medical University Huai'an Jiangsu China

**Keywords:** apoptosis, cell cycle, KIF20A, lung adenocarcinoma, proliferation, TCGA

## Abstract

Increasing studies showed that kinesin family member 20A (KIF20A) was overexpessed in several types of cancer, and its overexpression correlated with the oncogenesis and prognosis of cancers. However, little is known about the role of KIF20A in lung adenocarcinoma (LUAD). In this study, we employed the bioinformatics analysis to identify the upregulation of KIF20A in LUAD, then verified the results in human tumor specimens and LUAD cell lines. Compared with normal lung tissues, a ubiquitous upregulation of KIF20A was observed in LUAD tissues by immunohistochemistry (IHC) as well as TCGA analysis. Higher expression of KIF20A was significantly associated with more advanced clinicopathological features and shorter overall survival (OS). Moreover, multivariate Cox regression analysis revealed that KIF20A was an independent prognostic factor for OS. The expression of KIF20A was significantly elevated in LUAD cell lines. After silencing KIF20A, lung cancer cell cycle arrested in G1 phase and apoptosis increased. The same results were observed in vivo. Thus, our study demonstrated that KIF20A might confer malignant phenotype to LUAD by regulating cell proliferation and apoptosis, providing a new potential biomarker for clinical treatment of LUAD.

## 1 INTRODUCTION

Lung adenocarcinoma (LUAD), the most common subtype of lung cancer, is one of the leading causes of the highest cancer‐related morbidity and mortality worldwide.^1^ With the advancement of science, the treatment of LUAD is developing, including surgical treatment, radiotherapy, chemotherapy and molecular targeting therapy, however, the overall survival rate is not optimistic.^2^ Therefore, finding a novel biomarker with therapeutic potential for individualized treatment of LUAD is urgently warranted.

The Kinesin family (KIF), which was first identified in 1985, contains 14 super families ranged from kinesin‐1 to kinesin‐14.^3^ Kinesin family member 20A (KIF20A), known as MKLP2 and RAB6KIFL, locates on chromosome 5q31.2 and belongs to kinesin family‐6, sharing a highly conserved motor domain with other Kinesin family members.^4^ KIF20A was upregulated in malignant tumors but barely expressed in normal organs except the testis and thymus.^5^, ^6^ In 2005, for the first time, the oncogenic properties of KIF20A was confirmed in pancreatic cancer, and downregulating KIF20A could significantly decrease tumor cell proliferation.^6^ Then its carcinogenic traits were reported in various cancers, such as nasopharyngeal carcinoma,^7^ hepatocarcinoma^8^,pancreatic cancer^6^, ^9^,melanoma,^10^ and glioma.^11^, ^12^ Accordingly, KIF20A was considered a tumor‐associated antigen (TAA), and its overexpression correlated with the oncogenesis and prognosis of cancers. Cancer cells overexpressed KIF20A can be recognized by hosts to initiate immune response, thereby KIF20‐derived peptides could be used as a novel immunotherapy agent. Several clinical trials using KIF20A vaccine had been performed.^13^, ^14^, ^15^, ^16^, ^17^ However, no data to date are available about the role of KIF20A in LUAD.

In this study, for the first time we analysed the expression and function of KIF20A in LUAD. KIF20A was identified as one of the co‐upregulated genes among four independent lung cancer gene microarray datasets downloaded from the Gene Expression Omnibus (GEO) database. Both in vitro and in vivo experiments showed that KIF20A might confer malignant phenotype to LUAD by promoting cell proliferation and inhibiting apoptosis. Our results suggested that KIF20A could serve as a novel biomarker with therapeutic potential for treatment of LUAD.

## 2 MATERIALS AND METHODS

### 2.1 Bioinformatics analysis

A total of four lung cancer gene microarray datasets (GSE10072, GSE30219, GSE32863, and GSE 83213) were obtained from GEO database (http://www.ncbi.nlm.nih.gov/geo). The four gene expression profiles contained 58 LUAD samples and 49 para‐tumor samples,85 LUAD samples and 14 para‐tumor samples, 58 LUAD samples and 58 para‐tumor samples, 11 LUAD samples and 46 para‐tumor samples respectively. The differentially expressed genes (DEGs) were analysed using the limma package in R language. Then the co‐upregulated DEGs of the four gene expression profiles were identified with a Venn Diagram (http://bioinfogp.cnb.csic.es/tools/venny/index.html).

The Cancer Genome Atlas (TCGA; http://cancergenome.nih.gov) was used to explore the function of KIF20A in LUAD. The dataset contains a list of 513 LUAD samples and 59 adjacent normal tissue samples with complete clinical information. Kaplan‐Meier analysis was used to evaluate the prognostic value of KIF20A in LUAD patients. Gene set enrichment analysis (GSEA; http://www.broad.mit.edu/gsea) was carried out to discern gene sets changed by high KIF20A expression compared with low KIF20A expression from the public TCGA database.

### 2.2 Patients and samples

A total of 62 cases of formalin‐fixed paraffin‐embedded (FFPE) LUAD tissue samples had been diagnosed both clinically and histologically at First People's Hospital of Yancheng, Fourth Affliated Hospital of Nantong University (Yancheng, China) between 2012 and 2014. No patients received chemotherapy or radiotherapy before surgical operation. The study was approved by the Institutional Ethics Committee of First People's Hospital of Yancheng, and written informed consent was signed by each patient.

### 2.3 Immunohistochemistry

The FFPE sections were first deparaffinized in xylene, then rehydrated, followed by antigen retrieval using 0.01 M citrate buffer (pH 6.0). The section was then incubated with 3% H_2_O_2_ for 15 minutes to block endogenous peroxidase. Then, the sections were incubated with mouse anti‐human KIF20A monoclonal primary antibody (1:500, sc‐374508; Santa Cruz, USA) and mouse anti‐human Ki‐67 monoclonal primary antibody (1:500, sc‐23900; Santa Cruz, CA, USA) overnight at 4°C. After PBS washing, the sections were incubated with goat anti‐mouse biotinylated secondary antibody (Dako, Glostrup, Denmark) for 30 minutes at 37°C. Finally, the slide was developed with 3,3′‐diaminobenzidene (DAB) and then counterstained with hematoxylin.

The sections were independently scanned by two experienced pathologists. The percentage of positively‐stained cancer cells and the staining intensity were used to obtain a final staining score. The percentage of positively‐stained cancer cells was defined: 0 (no staining), 1 (1%‐25%), 2 (25%‐50%), 3 (50%‐75%) or 4 (75%‐100%). The staining intensity was defined: 0 (no staining), 1 (weak staining), 2 (moderate staining), or 3 (intense staining). The product of the intensity score and the percentage of cells stained was the final staining score which had a minimum value of 0 and a maximum of 12. A score ≥6 was considered high expression.

### 2.4 Cell lines and culture

Human LUAD cells H1975, A549 and HCC827 were purchased from Cell Bank, Shanghai Institutes for Biological Sciences, Chinese Academy of Sciences. PC9 cells and human bronchial epithelial cell (HBE) were kindly gifted by Dr. Chen (Nanjing Medical University, Nanjing, China). All cells were cultured in RPMI 1640 (Gibco, Carlsbad, CA, USA) supplemented with 10% fetal bovine serum (FBS; Gibco), 100 μg/μL streptomycin and 100 μg/μL penicillin in a humidified incubator at 37°C and 5% CO_2_.

### 2.5 Quantitative real‐time polymerase chain reaction (qRT‐PCR)

Total RNA was isolated from cultured cell lines using Trizol reagent (Invitrogen, Carlsbad, CA, USA). A final volume of 20 μL cDNA was synthesized from 10 μg total RNA using Reverse Transcription Kit (Takara, Biotechnology, Dalian, China). The primers were designed as follows: for KIF20A, forward primer, 5′‐TGCTGTCCGATGACGATGTC‐3′, and reverse primer, 5′‐AGGTTCTTGCGTACCACAGAC‐3′; for GAPDH, forward primer, 5′‐AGGTTCTTGCGTACCACAGAC‐3′, and reverse primer, 5′‐GCCATCACGCCACAGTTTC‐3′. Quantitative reverse‐transcriptase polymerase chain reaction (qRT‐PCR) analyses were performed using SYBR Green (Invitrogen) and the ABI prism 7300 Sequence Detection System (Applied Bio‐systems). Each sample was run in triplicate, and the relative expression was calculated and normalized using the 2^−ΔΔCt^ method relative to GAPDH.

### 2.6 Protein preparation and western blot

Cells cultured to 80%‐90% confluent were harvested and lysed in radio immunoprecipitation assay (RIPA; Invitrigon, USA) buffer added with 1% PMSF and 1% protease inhibitor cocktail (Boster, Wuhan, China) on ice. Protein concentrations were quantified using a BCA kit (Beyotime, Shanghai, China). Equal amounts of protein were separated by 10% SDS‐PAGE gel electrophoresis, then transferred to PVDF membranes. The membranes were blocked using 2% BSA in TBST (Beyotime, Shanghai, China) for 1.5 hour at room temperature. Membranes were incubated with primary antibodies against KIF20A (1:100,sc‐374508; Santa Cruz, CA, USA), Cyclin D1 (1:1000, 2922,CST, USA), Cyclin E1 (1:200,sc‐247; Santa Cruz, USA),p21 (1:1000, 2947,CST, USA),p27 (1:200,sc‐56338; Santa Cruz, USA) and actin (1:1000, 4970,CST, USA) overnight at 4°C. After being washed in TBST, membranes were incubated with 1:5000‐diluted a goat anti‐rabbit/mouse‐IgG secondary antibody conjugated with polymers of HRP (7074/7076, CST, USA) for 1 hour at room temperature. An ECL detection reagent (Beyotime, Shanghai, China) was used to visualize protein bands. Quantitative analyses of western blots were performed using Image J program (National Institutes of Health, Bethesda, MD, USA).

### 2.7 Small interfering RNA (siRNA) transfection

SiRNA transfections were performed using Lipofectamine RNAiMAX (Invitrogen) according to the manufacturer's instructions. KIF20A siRNAs were purchased from RiboBio (Guangzhou, China).The targeting sequences were as follows: si‐KIF20A‐1: 5′‐CTCCGAGATGAAATTTGCA‐3′;si‐KIF20A‐2: 5‐GGTCTGTGGTACGCAAGAA‐3′; and si‐KIF20A‐3: 5′‐GTCGTAGTTTCTCCCATGT‐3′. si‐control was used as a negative control.

### 2.8 Cell proliferation assays

The cell proliferation was evaluated via Cell Counting Kit‐8 and colony formation assays. For Cell Counting Kit‐8 assay, cells were seeded in 96‐well plates at a density of 3000 cells in 100 μL of medium per well 48 hour after transfection. At 0, 24, 48, 72 hour after cell adherence, the old medium was changed to RPMI 1640 without serum added with 10 μL Cell Counting Kit‐8 reagent (KeyGEN, Nanjing, China) per well. The absorbance was detected at wavelength 450 nm (450 OD) using a microplate reader followed by incubation at 37°C for 1 hour. For colony formation assays, 100 transfected cells were placed in six‐well plates and incubated in complete medium; the medium was exchanged twice a week. After 14 days, cells were fixed with 4% paraformaldehyde and stained with 0.1% crystal violet. Visible colonies were counted. All the experiments were conducted in triplicate.

### 2.9 Flow cytometry analysis

Flow cytometry analysis was performed to detect cell cycle distribution and cell apoptosis. For cell cycle analysis, cells were seeded in 6‐well plates. Cells were harvested 48 hours after transfection with corresponding si‐RNAs, washed twice with precooling PBS, fixed with ice‐cold 75% ethanol overnight at −20°C. Cell pellets were suspended in 1 mL of propidium iodide (PI) staining solution in the dark at 37°C for 10 minutes. Samples were analysed using a FACSCalibur flow cytometer (BD Biosciences, Franklin Lakes, NJ, USA). The percentage of cells in G0‐G1, S, and G2‐M phase were calculated. For apoptosis analysis, cells were treated with the same process as above‐mentioned. Then an Annexin V‐FITC Apoptosis Detection Kit (BD Biosciences) was used following the manufacturer's protocol. After incubation for 15 minutes at room temperature protected from light, the samples were rapidly analyzed by a FACScan flow cytometer (BD Biosciences). Each experiment was repeated three times. Both cell cycle and apoptosis were analyzed by FlowJo V10 software (TreeStar, Ashland, OR, USA).

### 2.10 Xenograft experiment

All animal experiments were conducted in accordance with the ethical standards and national guidelines and approved by Nanjing Medical University Animal Care Committee. To preparation of xenograft model, A549 cells suspensions (5 × 10^6^) were subcutaneously injected into the flanks of 12 female nude mice (6 weeks old). About 3 weeks after tumor inoculation, tumor volume reached 90 mm^3^ approximately, the mice were randomly divided into two groups for subsequent treatments. 5 nmol Cholesterol‐conjugated si‐KIF20A and corresponding si‐Control (RiboBio, Guangzhou, China) dissolved in 20ul saline were injected intratumorally at multiple points once every 3 days for six times.^18^ Tumor volume was estimated by calipers and calculated as *L* × *W* ^2 ^× 1/2 (*L*, length; *W*, width). After mice sacrificed, tumors were dissected and weighted, then embedded in paraffin for further analysis.

### 2.11 Statistical analysis

All statistical analysis was performed using SPSS 22.0 software (IBM, Armonk, New York, USA). Correlation between KIF20A expression and clinicopathologic parameters was assessed by chi‐square test. Correlation between KIF20A and Ki67 expression was examined by chi‐square test or Pearson's test. Overall survival (OS) was evaluated by Kaplan‐Meier survival curve and analyzed by the log‐rank test. Cox regression model was performed to identify the prognostic factors by univariable and multivariable analysis. The two groups were compared using independent Student's *t* test. Data were present as mean ± SD. Difference with *P* < 0.05 was considered statistically significant.

## 3 RESULTS

### 3.1 KIF20A was upregulated in LUAD

**Figure 1:**
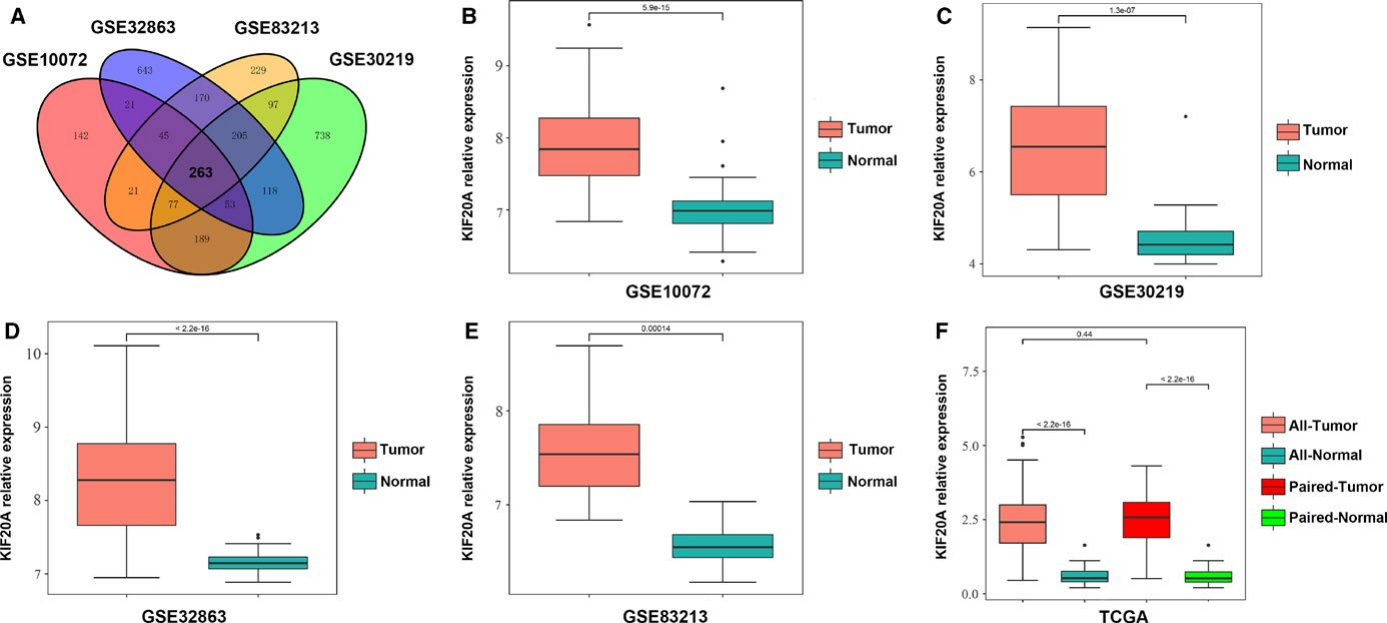
KIF20A was upregulated in LUAD tissues based on GEO and TCGA database. A, Venn diagram analysis of the overlapping up‐regulated genes in four independent GEO datasets. B‐E, KIF20A mRNA level of LUAD tissues and adjacent normal lung tissues in dataset GSE10072 (B, 58 tumor vs 49 para‐tumor), GSE30219 (C, 85 tumor vs 14 para‐tumor), GSE32863 (D, 58 tumor vs 58 para‐tumor), GSE83213 (E, 11 tumor vs 46 para‐tumor). F, KIF20A expression in TCGA dataset containing 513 LUAD tissues and 59 adjacent normal tissues (57 paired tissues)

To identify novel oncogenic genes in LUAD, we performed data‐mining analysis for microarray datasets (GSE10072, GSE30219, GSE32863, and GSE 83213) downloaded from the GEO database. We assessed the upregulated genes (FC > 1.5, *P *<* *0.01) in each data set, then screened out 263 overlapping genes among the four datasets (Figure 1A). Among these 263 co‐upregulated genes, we noted that there were several KIF genes, such as KIF4A, KIF20A, KIF11, and KIF2C. The roles of KIF4A, KIF11 and KIF2C in lung cancer have been already reported,^19^, ^20^, ^21^ while the involvement of KIF20A in LUAD remains largely unknown. The mRNA levels of KIF20A in LUAD was significantly upregulated than that in normal lung tissues in each dataset (all *P* < 0.001, Figure 1B‐E). To confirm the role of KIF20A in LUAD, we further analyzed the expression of KIF20A in the public TCGA database. The analysis showed that the mRNA levels of KIF20A in LUAD were significantly elevated compared with those of normal controls, and the results was consistent in paired samples (both *P* < 0.0001, Figure 1F).

### 3.2 Overexpression of KIF20A was correlated with malignant clinical features in LUAD

Given the high expression of KIF20A in LUAD, we further analyzed the relationship between KIF20A and clinicopathological factors. The correlation of KIF20A and clinicopathological features was validated in a large LUAD cohort with complete clinicopathological information from TCGA dataset (n = 464, patients without complete clinical information and follow‐up data were excluded). The results suggested that the expression of KIF20A in LUAD were related to age, gender, lymph node metastasis status, and tumor stage (Table 1).

**Table 1:** Correlation between KIF20A expression and clinicopathological features in LUAD patients in TCGA dataset

Clinicopathological features	Total 464	KIF20A‐high	KIF20A‐low	*P* value
Age (y)				
≥65	262	114	148	0.001^a^
<65	202	118	84	
Gender				
Female	248	112	136	0.026^a^
Male	216	120	96	
T classification				
T1‐2	404	200	204	0.580
T3‐4	60	32	28	
Lymph node metastasis				
Positive	160	98	62	0.001^a^
Negative	304	134	170	
Tumor stage				
I‐II	366	168	198	0.001^a^
III‐IV	98	64	34	

a Significant difference in statistics.

**Figure 2:**
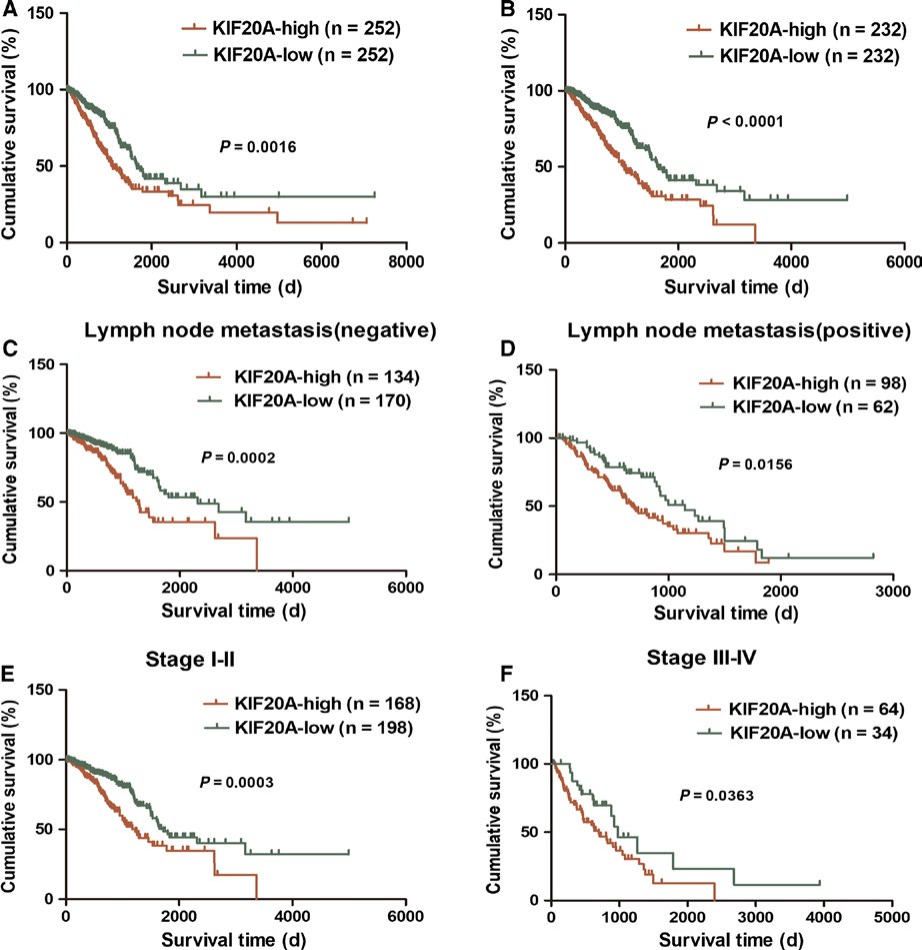
Kaplan‐Meier analysis of overall survival in LUAD patients from TCGA dataset. OS analysis of KIF20A expression in LUAD patients with follow‐up data (n = 504) (A) and cohort also with complete clinicopathological information (n = 464) (B). Subgroup analysis in lymph node nonmetastatic group (C), metastatic group (D), stage I‐II group (E) and stage III‐IV group (F)

Kaplan‐Meier survival analysis and log‐rank test were performed to estimate the clinical prognostic significance of KIF20A expression in LUAD patients with follow‐up data from TCGA. In LUAD patients with survival information, the overall survival (OS) of those with high KIF20A expression was shorter (OS) (*P* = 0.0016, n = 504, Figure 2A). Excluding patients with incomplete clinicopathological information, similar result was displayed (*P* < 0.0001, n = 464, Figure 2B). We further conducted subgroup analysis. The results showed that patients with high KIF20A expression had a shorter survival time in both the lymph node metastatic group and the nonmetastatic group (*P* = 0.0156 and 0.0002, respectively, Figure 2C‐D); whether in early or advanced groups, patients with elevated KIF20A expression had lower survival periods compared with patients with lower KIF20A expression (*P* = 0.0003 and 0.0363, respectively, Figure 2E‐F).

Moreover, univariate and multivariate Cox regression analysis were used to evaluate the role of KIF20A and other prognostic parameters in TCGA LUAD patients. In univariate analysis, higher KIF20A expression, positive lymph node metastasis, advanced T stage and TNM stage were significantly correlated with worse prognosis. Multivariable Cox regression analysis demonstrated that expression of KIF20A (HR = 1.782, 95% CI: 1.296‐2.451, *P* < 0.001), T stage (HR = 1.737, 95% CI: 1.137‐2.652, *P* = 0.011) and lymph node metastatic status(HR = 1.968, 95% CI: 1.370‐2.825, *P* < 0.001), were related to OS (Table 2).In conclusion, high expression of KIF20A can be considered as an independent risk factor of OS in patients with LUAD.

**Table 2:** COX regression analyses of overall survival in LUAD patients in TCGA dataset

Prognostic parameter	Univariate analysis			Multivariate analysis		
	HR	95%CI	*P* value	HR	95%CI	*P* value
Age (≥65 vs <65)	1.018	0.750‐1.381	0.910	‐	‐	‐
Gender (Female vs Male)	0.853	0.631‐1.151	0.298	‐	‐	‐
T classification (T1‐2 vs T3‐4)	2.170	1.480‐3.182	<0.001	1.737	1.137‐2.652	0.011^a^
Lymph node metastasis (Negative vs Positive)	2.509	1.852‐3.398	<0.001	1.968	1.370‐2.825	<0.001^a^
Tumor stage (I‐II vs III‐IV)	2.355	1.709‐3.246	<0.001	1.200	0.793‐1.818	0.388
KIF20A expression(Low vs High)	2.120	1.554‐2.891	<0.001	1.782	1.296‐2.451	<0.001^a^

a Significant difference in statistics.

### 3.3 Expression of KIF20A was associated with Ki‐67 levels in LUAD tissues

**Figure 3:**
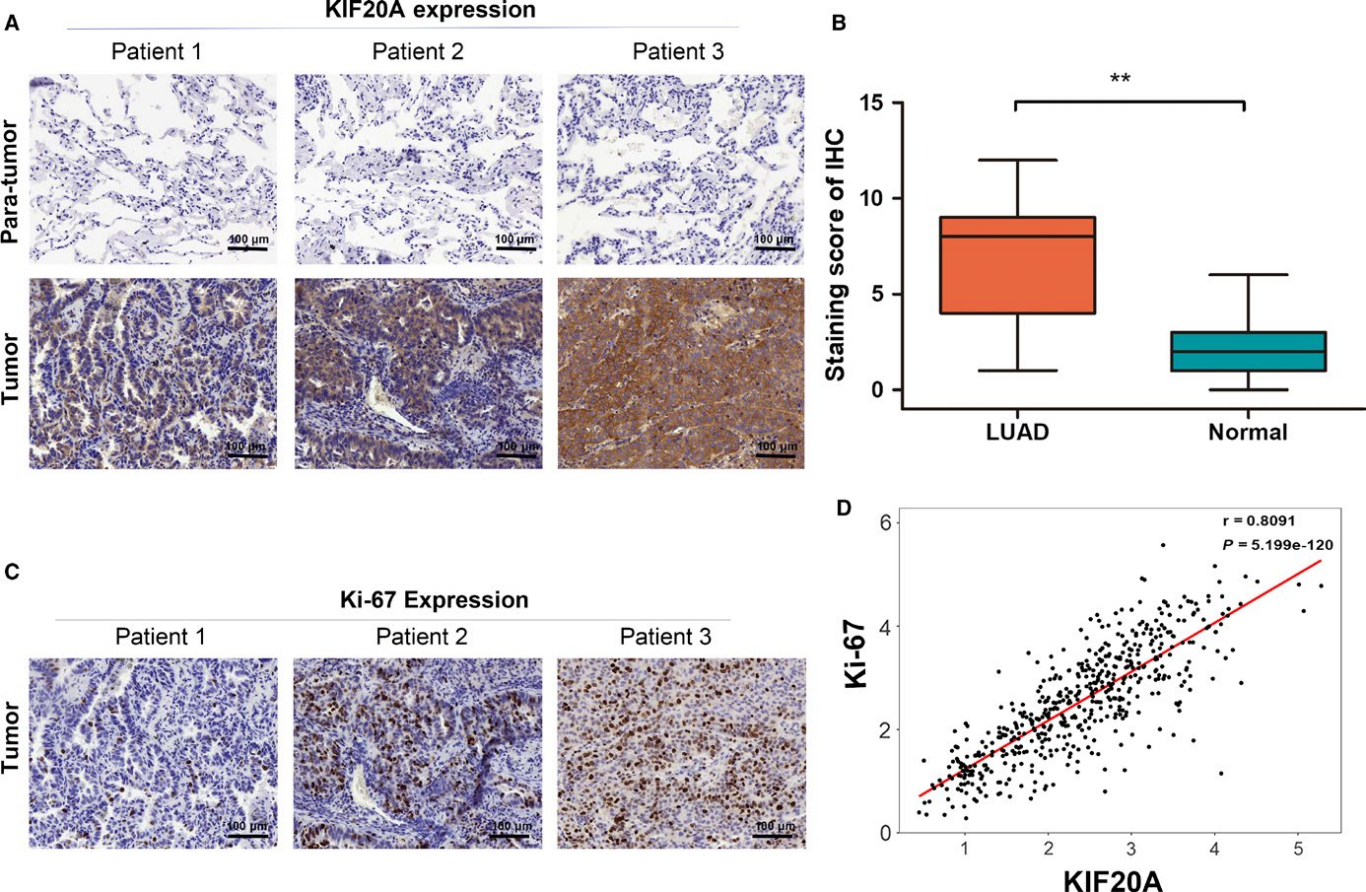
Immunohistochemistry analysis of KIF20A and correlation with Ki‐67. A, Representative IHC images of KIF20A in clinical LUAD tissues and adjacent normal lung tissues. B, IHC staining score of KIF20A in LUAD tissues and normal lung tissues (n = 62). C, IHC images of Ki‐67 in the same clinical LUAD tissues with A. D, The Pearson's correlation analysis between KIF20A and Ki‐67 expression in TCGA LUAD samples (n = 513).Objective 20×, Scale bar, 100 μm. **P < 0.01

The results of bioinformatics analysis showed that KIF20A was an oncogene in lung cancer. Based on this result, we proposed whether KIF20A was related to the proliferation of lung cancer and affected the proliferation of lung cancer cells. Immunohistochemistry was performed to evaluate the expression of KIF20A in 62 pairs of LUAD and adjacent normal tissues. As shown in Figure 3A, KIF20A was mainly located in the cytoplasm and membranes. There was absent or weak staining of KIF20A in normal lung tissues, whereas, a ubiquitous positive staining of KIF20A was observed in LUAD tissues. The KIF20A staining scores in LUAD tissues were significantly higher than that in adjacent normal tissues (*P* < 0.0001, Figure 3B), consistent with the mRNA expression mentioned above (Figure 1B‐F).

We also performed the staining of Ki‐67 on all these LUAD samples, which is confirmed as an indicator for tumor proliferation. As shown in Figure 3C, there were generally positive expression of Ki‐67 in LUAD tissues which mainly located in the nucleus of tumor cells. The association of KIF20A and Ki‐67 expression in LUAD tissues was evaluated by chi‐square test, showing that the levels of KIF20A was positively correlated with the expression of Ki‐67 (*χ* ^2^ = 18.502, *P* < 0.001, Table 3), which was consistent with the Pearson's correlation analysis of the two factors in LUAD tissues from TCGA dataset (*r* = 0.809, *P* < 0.001, n = 513, Figure 2D).

**Table 3:** Association of KIF20A and Ki‐67expression in LUAD samples

	Ki‐67 expression		*P* value
	High	Low	
KIF20A expression			
High	30	10	<0.001^a^
Low	4	18	

a Significant difference in statistics.

### 3.4 Knockdown of KIF20A inhibited LUAD cells proliferation and induced cell apoptosis

**Figure 4:**
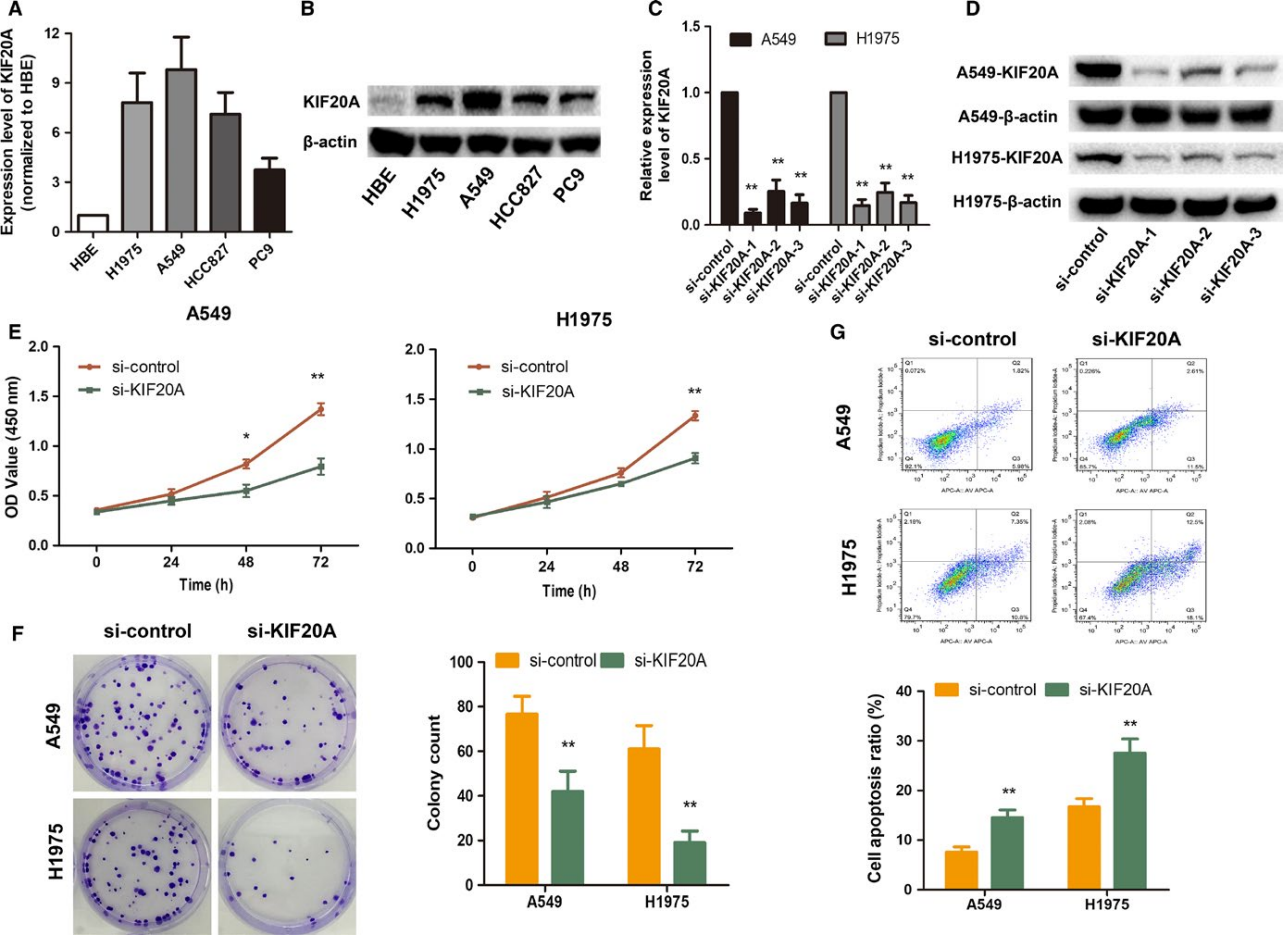
Knockdown of KIF20A inhibited LUAD cells proliferation and induced cell apoptosis. KIF20A expression in HBE and four LUAD cells at mRNA (A) and protein level (B). A549 and H1975 cell lines with highest KIF20A expression were chosen for further investigation. Si‐RNA transfection technology was used to silence KIF20A expression and the transfection efficiency was verified by qRT‐PCR (C) and WB (D).Si‐KIF20A‐1 was chosen for subsequent study. Cell proliferative activity was measured after knockdown of KIF20A by CCK8 (E) and colony formation assays (F). Cell apoptosis rate was assessed after downregulation of KIF20A by Flow cytometry analysis (G). *P < 0.05, **P < 0.01

We further validated effects of KIF20A on malignant biological behavior of LUAD in vitro. First, we explored the differential expression of KIF20A among four LUAD cell lines and HBE cells. As shown in Figure 4A,B, compared with HBE cells, KIF20A expression in the four LUAD cell lines was significantly increased at both mRNA and protein levels, and the highest expression of KIF20A was obtained in A549 and H1975 cell lines, which were chosen for further study. Next, we suppressed the expression of KIF20A using si‐RNA transfection technology and verified the transfection efficiency by qRT‐PCR and Western Blot assays. As shown in Figure 4C,D, si‐KIF20A‐1 with the best inhibited efficiency was thereby chosen for subsequent cell phenotype study. CCK8 and colony formation assays both showed that cell proliferative activity was significantly reduced after knockdown of KIF20A (Figure 4E,F). We also examined the effect of KIF20A on cell apoptosis by flow cytometry analysis. As shown in Figure 4G, the apoptosis rate of si‐KIF20A transfected LUAD cells was significantly higher than that of control group.

### 3.5 Silencing KIF20A arrested the cell cycle of LUAD cells

**Figure 5:**
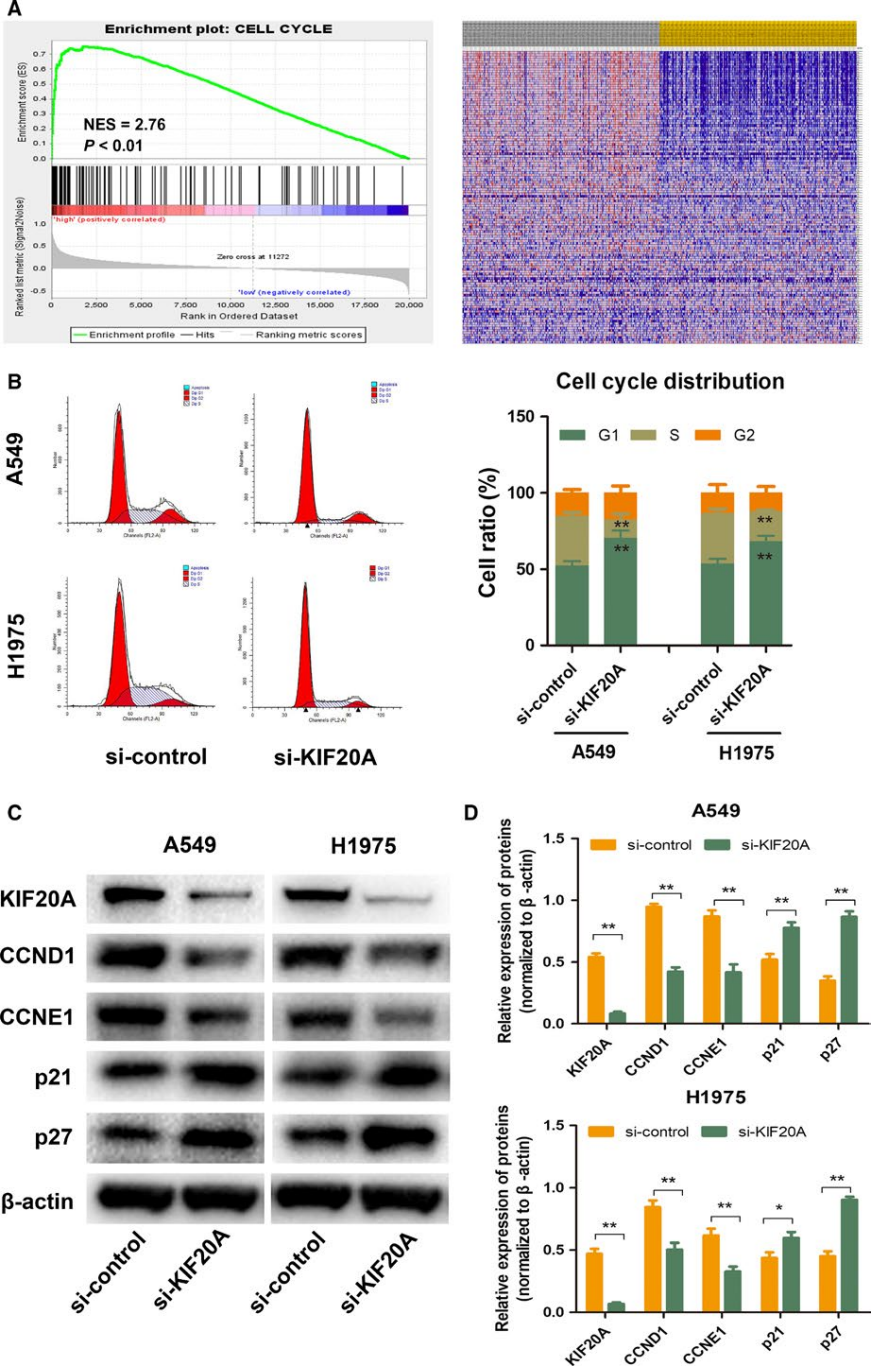
Knockdown of KIF20A lead to cell cycle arrest in LUAD cells. (A) The top pathway enriched by GSEA in TCGA dataset by comparing the KIF20A‐high and KIF20A‐low expression samples was ‘CELL‐CYCLE’ pathway. NES = normalized enrichment score. (B) Cell cycle distribution was analyzed by Flow cytometry analysis in A549 and H1975 cell lines with KIF20A knockdown or not. The expression of G1 phase related genes was detected by WB (C) and quantified by grayscale values (D). *P < 0.05, **P < 0.01

To explore the potential mechanism of KIF20A involved in the malignant biological behavior of LUAD, we employed gene set enrichment analysis (GSEA) using TCGA dataset. As shown in Figure 5A, the top pathway related to overexpressed KIF20A in LUAD was ‘CELL CYCLE’. Thus, we examined the effect of KIF20A on cell cycle by flow cytometry analysis. As shown in Figure 5B, after knocking down of KIF20A, the percentage of LUAD cells in G1 phase significantly increased, while the percentage of cells in S phase markedly decreased, indicating that downregulation of KIF20A could arrest lung cancer cells in G1 phase. To further confirm the connection between KIF20A expression and cell cycle in LUAD, we detected the protein expression levels of G1 related genes by WB and quantified them by grayscale values, including Cyclin D1(CCND1), Cyclin E1(CCNE1), p21 and p27. As shown in Figure 5D,E, after silencing of KIF20A, the expression of CCND1 and CCNE1 was markedly decreased compared with the control group, while the expression of p21 and p27 was significantly increased.

### 3.6 Knockdown of KIF20A inhibited tumor growth in vivo

**Figure 6:**
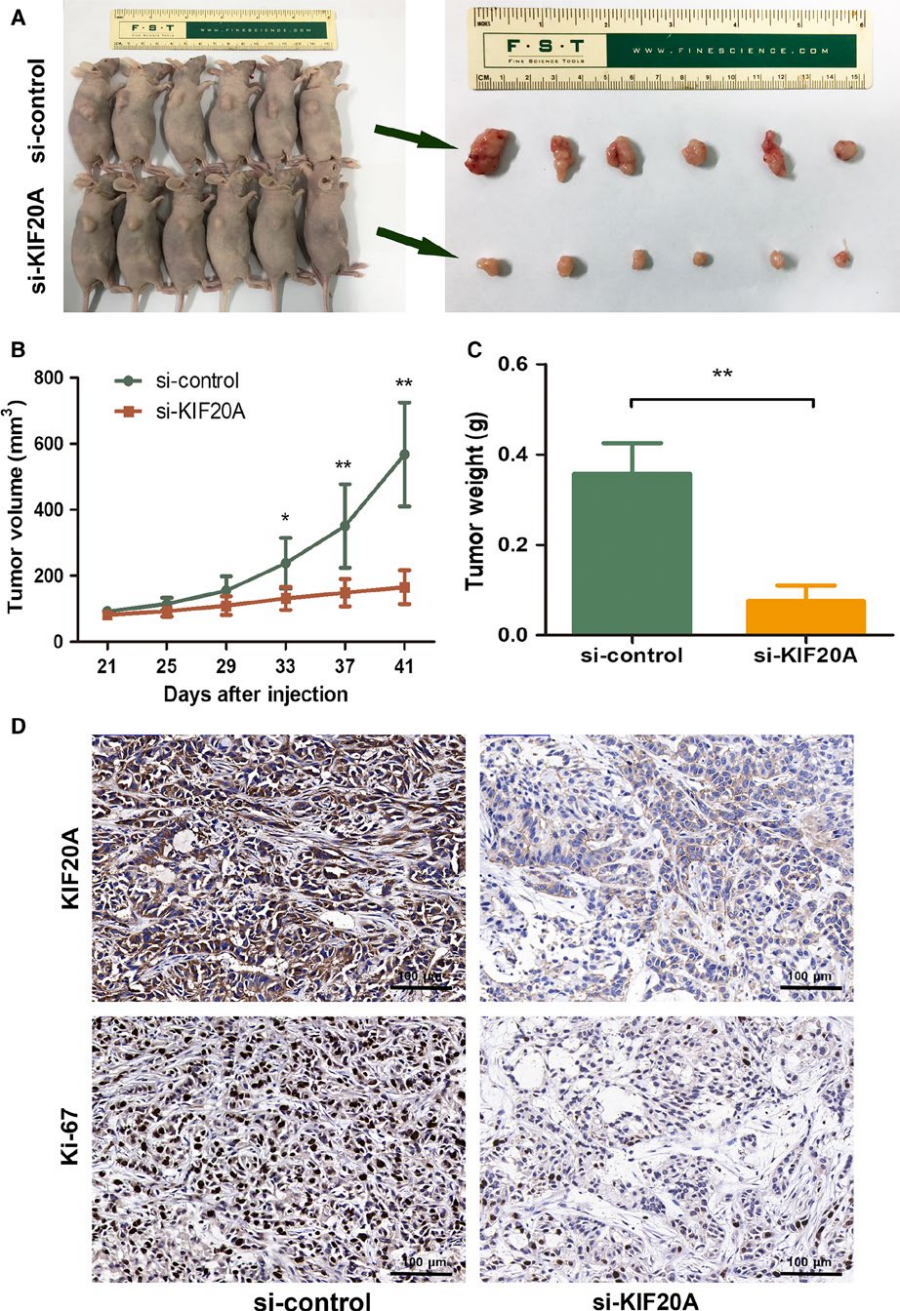
Knockdown of KIF20A inhibited tumor growth in vivo. A‐B, Tumor volumes of A549 cell subcutaneous xenograft were compared between si‐KIF20A and si‐control group. C, Tumor weights were compared between si‐KIF20A and si‐control group. D, Representative IHC images of KIF20A and Ki‐67 in xenograft tumors. *P < 0.05, **P < 0.01

To evaluate the tumorigenicity of KIF20A in vivo, A549 cell line was used to establish xenograft tumor models. Three weeks after tumor inoculation, the mice were injected with cholesterol‐conjugated si‐KIF20A and corresponding si‐control, respectively. As shown in Figure 6A,B, tumor volumes of si‐KIF20A group were significantly reduced compared with those of si‐control group. Knockdown of KIF20A could significantly decrease tumor weight (Figure 6C). Using IHC analysis, we confirmed KIF20A was silenced in si‐KIF20A group. Ki‐67 staining showed the similar result that tumors with highly expressed KIF20A had increased levels of Ki‐67(Figure 6D). These data revealed that KIF20A could promote tumor growth in vivo.

## 4 DISCUSSION

KIF20A is a member of the Kinesin superfamily containing a conserved motor domain which binds to microtubules to generate the energy required for protein movement.^3^ KIF20A was first reported to interact with Rab6 small GTPase and involved in the dynamics of the Golgi apparatus.^4^It accumulated in mitotic cells and participated in formation of the mitotic spindle, thereby playing an important role in cytokinesis.^22^Aberrant expression of KIFs could lead to spindle defects, chromosomal abnormal distribution and aneuploidy, which is closely related to tumorigenesis.

It have been demonstrated that KIF20A was overexpressed in multiple tumors and was associated with tumorigenesis and prognosis. In this study, for the first time we confirmed the oncogenic role of KIF20A in LUAD. At mRNA level, we applied bioinformatics methods to further validate the role of KIF20A in LUAD. Consistent with the KIF20A level in GEO databases, TCGA analysis suggested that the expression of KIF20A in LUAD was significantly increased than that in normal lung tissues. Based on those above findings, we speculated whether levels of KIF20A were associated with clinical characteristics and prognosis in patients with LUAD. We next performed analyses in a large cohort with LUAD from TCGA dataset. Correlation analyses about KIF20A expression and clinicopathologic parameters revealed that KIF20A expression correlated with age, gender, lymph node metastatic status and TNM stage. For prognostic value analyses, as expected, Kaplan‐Meier survival analyses revealed that patients with higher expression of KIF20A had shorter OS regardless of lymph node metastatic status and TNM stage. In addition, univariate as well as multivariate Cox regression analyses demonstrated that KIF20A expression can be considered as an independent prognostic factor of OS in patients with LUAD. Our findings were highly consistent with previous studies about the prognostic impact of KIF20A in other cancers.^7^, ^8^, ^11^, ^23^

At protein level, we observed a ubiquitous upregulation of KIF20A in LUAD tissues compared with normal lung tissues by IHC. As a confirmed tumor proliferation index, Ki‐67 was widely used as an indicator for the prognosis in clinical practice of non‐small cell lung cancer.^24^, ^25^ Using chi‐square test, we analyzed the correlation between KIF20A expression and Ki‐67 in LUAD tissues. Our result showed that KIF20A expression in LUAD was highly positively correlated with Ki‐67 level, bearing similarities with observations from Duan et al^11^ who reported that the expression of KIF20A was relevant to Ki67 expression in gliomas. The Pearson's correlation analysis of the two factors in LUAD tissues from TCGA dataset was consistent with our findings, implying that KIF20A could be an indicator of tumor proliferation as well as Ki‐67.

To further illustrate the biological function of KIF20A in LUAD, we explored the expression of KIF20A in different LUAD cell lines. Consistent with the clinical results, KIF20A expression in LUAD cells was significantly elevated compared to HBE cells. The in vitro results revealed that knockdown of KIF20A could drastically attenuate LUAD cells proliferation and induce tumor cells apoptosis. Dysregulation of cell proliferation and inhibition of apoptosis are core changes in the development of all tumors.^26^To explore the potential mechanism by which KIF20A drives LUAD tumorigenesis, we performed GSEA using the public TCGA dataset. GSEA data suggested that ‘CELL‐CYCLE’ pathway was enriched in patients with KIF20A‐higher expression. To determine whether knockdown of KIF20A inhibit cell proliferation through affecting cell cycle distribution, flow cytometry was performed for cell cycle analysis. Our experiments confirmed that downregulation of KIF20A lead to G1 phase arrest. Cell cycle dysregulation is one of the symbols of cancer.^27^ Moreover, G1/S phase transition is a main checkpoint in the cell cycle progression.^28^To further confirm the interconnection between KIF20A and cell cycle, we examined the expression of several G1 phase related genes, including CCND1, CCNE1, p21 and p27. Cyclins function as regulators of cell cycle. CCND1 forms a complex with and functions as a regulatory subunit of cyclin‐dependent kinase 4 (CDK4) or CDK6, whose activity is required for cell cycle G1/S transition.^29^ While CCNE1 regulates cell cycle G1/S transition by forming a complex with CDK2.^30^ p21, also named cyclin dependent kinase inhibitor 1A (CDKN1A), binds to and inhibits the activity of CDK2 or CDK4 complexes, and thus functions as a regulator of cell cycle progression at G1 phase.^31^ p27, also named cyclin dependent kinase inhibitor 1B (CDKN1B), shares some similarity with CDKN1A/p21. The encoded protein binds to and prevents the activation of cyclin E‐CDK2 or cyclin D‐CDK4 complexes, thereby controlling cell cycle progression in the G1 phase.^32^ Taken together, CCND1 and CCNE1 can promote G1‐S transition,which can be blocked by p21 and p27. In the present study, we found that after KIF20A silencing, the expression of CCND1 and CCNE1 was significantly decreased, while the expression of p21 and p27 was significantly increased. Hence, we concluded that KIF20A can affect cell proliferation by modulating the G1/S phase transition of LUAD cells. Finally, the subcutaneous xenograft model further identified that KIF20A could promote tumor growth in vivo, consistent with the in vitro result.

In the current study, there are also some limitations. Due to partial loss of follow‐up, clinical cases were not able to be carried out survival analysis. In the future work, we will expand the sample size, strengthen follow‐up and conduct detailed analyses.

To sum up, by data mining from public database, our study revealed that KIF20A was an upregulated gene in LUAD. KIF20A overexpression correlated with more advanced clinicopathological features and was an independent prognostic factor for OS. KIF20A suppression could inhibit LUAD cell proliferation via G1 phase arrest and induce apoptosis. The suppression of proliferation was also confirmed in vivo. Thus, our study demonstrated that KIF20A might confer malignant phenotype to LUAD by regulating cell proliferation and apoptosis, providing a novel biomarker with therapeutic potential for treatment of LUAD.

## CONFLICT OF INTEREST

The authors declare no conflicts of interest in this work.
